# Editorial: Challenges to research on oral potentially malignant disorders and oral cancer

**DOI:** 10.3389/fonc.2025.1639596

**Published:** 2025-06-23

**Authors:** Alberto Rodríguez-Archilla, Luis Alberto Gaitán-Cepeda

**Affiliations:** ^1^ Department of Stomatology, Oral Medicine Unit. Faculty of Dentistry, University of Granada, Granada, Spain; ^2^ Biohealth Research Institute (IBS), Granada, Spain; ^3^ Department of Oral and Maxillofacial Medicine and Pathology, Research and Graduate Division, Dental School, National Autonomous University of Mexico, Mexico City, Mexico

**Keywords:** early detection of cancer, mouth neoplasms, oral medicine, pathology oral, precancerous conditions

Oral squamous cell carcinoma (OSCC) is one of the most prevalent and lethal malignancies among patients with head and neck cancer. Despite advances in identifying risk factors, improved screening methods, and increasing interest in early detection, the global burden of oral cancer continues to grow. Central to this challenge are the complexities surrounding oral potentially malignant disorders (OPMDs), the heterogeneity of OSCC progression, and the multifaceted demands of patient care, particularly in long-term survivorship. In this Research Topic, four original research articles address key aspects of OSCC, including cause-of-death analysis, prognostic biomarker assessment, artificial intelligence (AI)-driven diagnostics, and surgical strategy evaluation. These studies underscore the challenges that researchers and clinicians face in understanding, detecting, and managing oral cancers.

A population-based study by Jiang et al. provided a compelling analysis of the causes of death among more than 30,000 patients with oral cancer using the SEER database. Notably, 27% of deaths were found to be due to non-cancer causes, with cardiovascular disease being the most common cause. This proportion rose to nearly 58% of patients followed for over 10 years. These findings reveal a critical gap in long-term cancer care: the need for comprehensive, multidisciplinary survivorship strategies. As OSCC survival improves, greater attention must be paid to managing chronic comorbidities, mitigating late treatment effects, and providing holistic care beyond oncological control.

In the field of prognostic biomarkers, Richter et al. examined the combined expression of p16^INK4a and MIB/Ki-67 in OSCC. While p16^INK4a has been widely studied, its prognostic value remains debated, often due to a lack of integration with proliferation markers such as Ki-67. This study identified a tumor subgroup with p16^INK4a positivity and low Ki-67 expression that was associated with significantly better five-year survival (83%) and improved recurrence-free and overall survival. These findings highlight the importance of combining biomarkers for risk stratification. Standardizing biomarker panels and validating them in multicenter studies will be essential for clinical translation.

Another frontier in OSCC research is the development of accurate and reproducible diagnostic tools.Li presented an innovative AI-based hybrid model that integrates Cross-Attention Vision Transformer (CrossViT) features with manually extracted pathological features for classifying histological images. When tested on two independent datasets, the model achieved diagnostic accuracies exceeding 99%, outperforming both CNN- and ViT-based methods. This approach enhances diagnostic precision and opens new pathways for computational pathology. However, key challenges remain, particularly regarding model generalizability across populations, the interpretability of AI decisions, and integration into routine diagnostic workflows.

From a surgical perspective, Vollmer et al. provided a timely re-evaluation of lymph node dissection strategies for primary OSCC. Their retrospective analysis compared supraomohyoid selective neck dissection (Levels I–III) with a more extensive dissection involving Levels IV and V. While broader dissections were linked to longer hospital stays, no significant survival benefit was observed. These results challenge the assumption that more extensive surgery inherently improves outcomes and instead advocate for personalized, anatomy-guided interventions. Such an approach may reduce morbidity while preserving oncological safety.

Taken together, these studies highlight several cross-cutting challenges in oral cancer research:

1. Long-Term Outcomes and Survivorship: As survival improves, the focus must shift from short-term oncological endpoints to comprehensive survivorship strategies. This includes addressing non-cancer mortality, late treatment effects, and long-term care through integrated clinical and policy approaches.
2. Biomarker Validation and Risk Stratification: OSCC heterogeneity demands more refined prognostic tools. Combinations of markers, such as p16^INK4a and Ki-67, show promise but they require harmonization and large-scale validation. Ultimately, these tools can support individualized treatment decisions.
3. Artificial Intelligence and Digital Pathology: AI’s diagnostic potential in OSCC is clear, but its widespread adoption faces barriers, including limited dataset diversity, algorithm transparency, and the need for clinician training. Bridging the gap between model development and clinical applications is a critical next step.
4. Surgical Optimization: Striking a balance between oncologic completeness and procedural morbidity is essential. Evidence-based tailored surgical strategies guided by lymph node mapping and tumor biology can provide better patient-centered outcomes.

Advancing OPMD and OSCC research will require a transdisciplinary approach that integrates molecular science, digital innovation, epidemiology, and clinical expertise. The studies presented in this Research Topic offer valuable insights and raise key questions: How can AI models be validated and implemented ethically in clinical practice? What infrastructure is needed to support the lifelong surveillance of oral cancer survivors? How can biomarker strategies be refined to enable personalized therapy?

These questions are central to the future of translational research and patient-centered care. By embracing these challenges, the oral oncology community can move closer to achieving more precise, equitable, and effective interventions.

